# Supplementary material for: Unveiling new quantum phases in the Shastry-Sutherland compound SrCu2(BO3)2 up to the saturation magnetic field
Source: Nat Commun. 2023 Jun 24;14:3769. doi: 10.1038/s41467-023-39502-5 (PMC10290659; doi:10.1038/s41467-023-39502-5)
Supplement: Supplementary file 1 — Supplementary Information [file 41467_2023_39502_MOESM1_ESM.pdf]

Supplementary Information for "Unveiling new quantum phases in the Shastry-Sutherland compound $SrCu_2(BO_3)_2$ up to the saturation magnetic field"

T. Nomura,[1,2,*] P. Corboz,[3,†] A. Miyata,[4] S. Zherlitsyn,[4] Y. Ishii,[1] Y. Kohama,[1] Y. H. Matsuda,[1] A. Ikeda,[5] C. Zhong,[6,‡] H. Kageyama,[6] and F. Mila[7]

[1]*Institute for Solid State Physics, University of Tokyo, Kashiwa, Chiba 277-8581, Japan*

[2]*Tokyo Denki University, Adachi, Tokyo 120-8551, Japan*

[3]*Institute for Theoretical Physics and Delta Institute for Theoretical Physics, University of Amsterdam, Science Park 904, 1098 XH Amsterdam, The Netherlands*

[4]*Hochfeld-Magnetlabor Dresden (HLD-EMFL), Helmholtz-Zentrum Dresden-Rossendorf, 01328 Dresden, Germany*

[5]*Department of Engineering Science, University of Electro-Communications, Chofu, Tokyo 182-8585, Japan*

[6]*Graduate School of Engineering, Kyoto University. Nishikyouku, Kyoto 615-8510, Japan*

[7]*Institute of Theoretical Physics, Ecole Polytechnique Fédérale de Lausanne (EPFL), 1015 Lausanne, Switzerland*

(Dated: June 6, 2023)

SUPPLEMENTARY NOTE 1: EXPERIMENTAL DETAILS

In this study, we used two types of pulsed magnets, the single-turn coil (STC) in the ISSP, University of Tokyo and the dual-coil pulsed magnet at the HLD in Dresden. Supplementary Fig. 1 shows the typical waveforms of these magnets. The STC is a semi-destructive technique where the field duration is limited to several μs. Because of this limitation, special experimental efforts are needed to measure the properties of materials in STC experiments. In this section, we discuss the experimental technique and present some further results.

For studying the ultrasonic properties up to 150 T, we used the continuous-wave (CW) excitation technique [1]. The experimental setting [Supplementary Fig. 2(a)] is very similar with the conventional pulse-echo technique, where the sample is sandwiched by two $LiNbO_3$ transducers. In the CW technique, the transducer is excited continuously without pulse modulation. Therefore, the ultrasound signal is also continuously detected even in the μs timescale. However, the detected signal includes cross-talk and many reflections, which affects the phase and amplitude of the acoustic waves. As discussed in Ref. [1], this effect becomes negligibly small if the sample length and shape are optimized. The relative change of the sound velocity $\Delta v/v_0$ is proportional to the relative change of the phase $\Delta\Phi/\Phi_0$ as

$$\Delta v/v_0 = -\Delta\Phi/\Phi_0 = -\Delta\Phi/(2\pi f \tau_0), \tag{1}$$

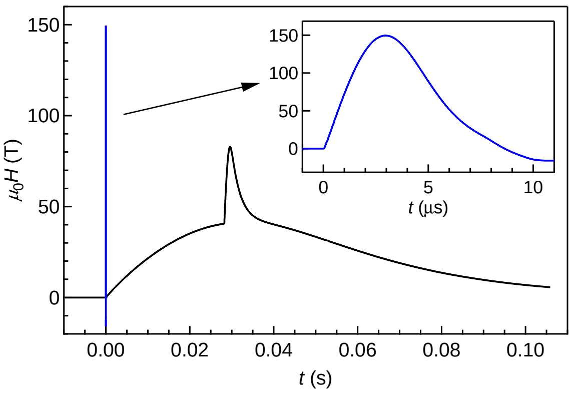

Supplementary Figure 1. Magnetic-field waveforms by the single-turn coil (blue, inset) and the dual-pulse magnet (black).

* tnomura@mail.dendai.ac.jp
† P.R.Corboz@uva.nl
‡ Present address: Department of Applied Chemistry, Ritsumeikan University, Kusatsu, Shiga 525-8577, Japan

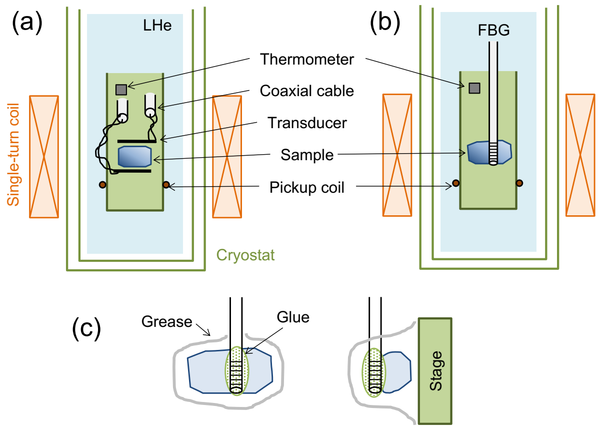

Supplementary Figure 2. Schematic settings of the STC (a) ultrasound and (b) magnetostriction experiments. (c) FBG setting view from two sides.

where f and τ_0 are the ultrasound frequency and the time delay of the ultrasound for a single transmitted signal. In this study, we first tested the installation up to 40 T in the STC and confirmed that the obtained data are consistent with the pulse-echo results. Here, we used the sample length of 1.1 mm, with which the time delay of the ultrasound is reasonably small.

Supplementary Fig. 3 shows the raw ultrasound data up to 125 T obtained at 3.2 K. The magnetic field profile is shifted by taking into account the propagation time of the ultrasound [from blue to black in Supplementary Fig. 3(a)]. The detected signal is shown in Supplementary Fig. 3(b). By applying the numerical lock-in, the phase shift ($\Delta\Phi$) and amplitude of the signal are extracted as shown in Supplementary Fig. 3(c) and Supplementary Fig. 3(d), respectively. Anomalies related to the phase transitions are observed both in the field-up and field-down sweeps. Note, that the detected phase has always $\pm 2\pi$ ambiguity. It means that if the phase change is very large and fast, the correct phase shift might be overlooked. This might be specifically the case at the 2/5-1/2 plateau region ($\sim$ 80 T, highlighted by light green), where the sound velocity changes by -40% per 5 T. Such a drastic sound velocity change is very challenging to follow in the STC experiment, because the sound velocity changes significantly within the sound propagation time even for 1.1 mm sample. Therefore, we rely on the results obtained in the non-destructive pulsed magnet by the conventional pulse-echo technique, and shifted accordingly the STC results in this field range [Supplementary Fig. 3(c)].

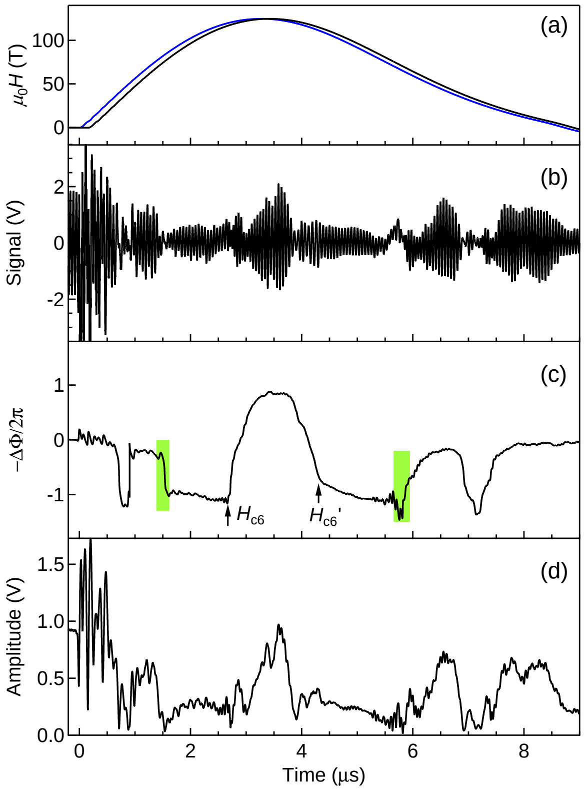

Supplementary Figure 3. Raw data of the ultrasound experiment for the c_{66} mode at 3.2 K up to 125 T. (a) Magnetic-field profile. The black curve is shifted from the original profile (blue) by taking into account the ultrasound-propagation time. (b) Detected ultrasound signal. (c) Phase change and (d) amplitude of the detected signal. The exact phase change is challenging to extract in the regions highlighted by light green because of the rapid change of the sound velocity in a very narrow magnetic field range.

For studying the magnetostriction, we used the fiber-Bragg grating (FBG) [2]. The schematic setting is shown in Supplementary Fig. 2(b). The FBG is fixed to the sample, and the sample deformation is monitored by the wavelength of the reflected light. By using the optical filter, the wavelength shift is detected as the amplitude change of the reflected light (for detail, see Ref. [2]). With this technique, the sample length change can be optically detected, which is a great advantage in the STC experiments. The biggest challenge performing the FBG measurement with STC is the sample vibration [3, 4]. When a magneto-structural phase transition occurs, a slight lattice deformation triggers a vibration of a crystal. When the sample shape is regular with parallel surfaces, this vibration results

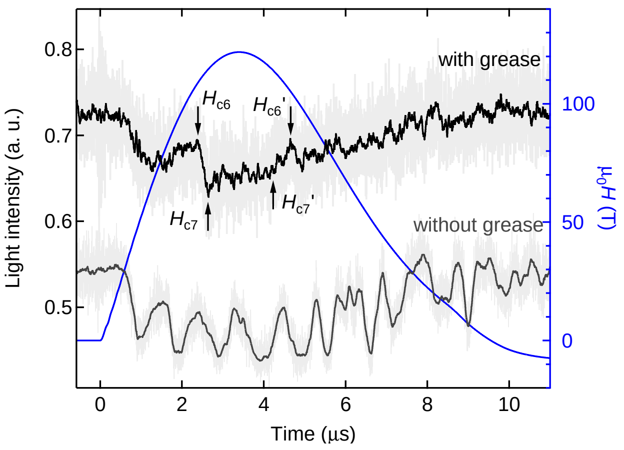

Supplementary Figure 4. Raw magnetostriction data (ΔL_c) at 4.2 K up to 122 T. Reflected light intensity (left) and magnetic field (right, blue curve) are plotted as a function of time. The results with and without grease are compared. The results (black curve) are averaged with the time window of 40 ns.

in the resonant oscillation of the crystal. Such oscillations lead to a periodic noise with the resonant frequency determined by the sample dimensions and the sound velocity. To attenuate such oscillations, we used (i) the sample with irregular shape and (ii) grease covering the whole sample [Supplementary Fig. 2(c)]. To weaken the resonant oscillation, the quality factor of the resonance has to be reduced. In this study, we used the crystal with one polished surface, but without any parallel surfaces. For such crystal, the phases of the reflected acoustic waves are random, and the resonant oscillations are strongly damped. Additionally, with vacuum grease, the sample is mechanically coupled with the sample stage. In this case, the quality factor of the resonance is strongly reduced by the mass-loading effect, and the resonant frequency becomes much lower than the STC characteristic frequency. Supplementary Fig. 4 shows the results of the magnetostriction experiment with and without vacuum grease. The resonant oscillations are strongly damped by the grease, although the size of the anomaly is also reduced. Therefore, in this study, we do not discuss the magnitude of the magnetostriction and only focus on the critical fields. The anomalies at H_{c6} and H_{c7} are well reproduced for the field-up and field-down sweeps.

Supplementary Fig. 5 summarizes the maximum-field dependence of the results. Supplementary Fig. 5(a) shows the phase of the ultrasound signal of the c_{66} mode. As discussed, the rapid change of the sound velocity is challenging to capture by this technique, which is

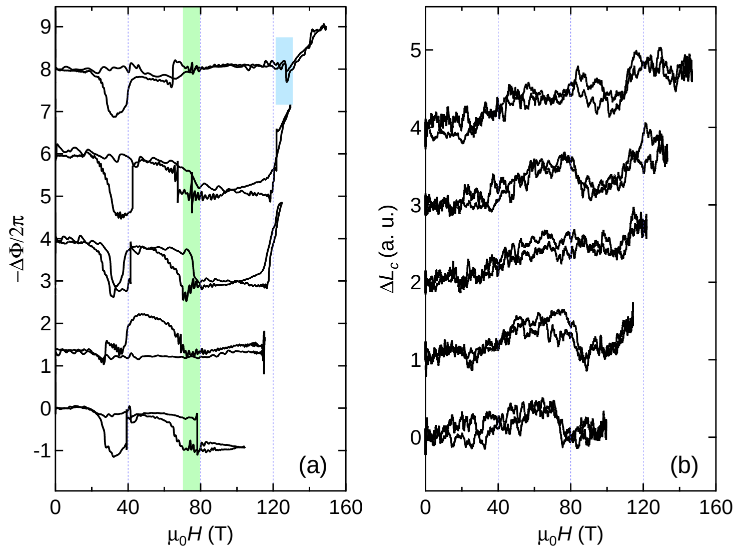

Supplementary Figure 5. (a) Phase shift of the acoustic wave for the c_{66} mode at 3.2 K and (b) magnetostriction along the c axis at 4.2 K measured to various maximum fields in the STC. The curves are deliberately shifted along the vertical axis for clarity. It is very challenging to reconstruct correctly the phase change in the green and blue area because of the very large and fast changes in the sound velocity in this magnetic field region.

the case in the region highlighted by light green and blue. Nevertheless, the overall features of the sound velocity are well reproduced; the softening in the 1/4 plateau, the hardening in the 1/3 plateau, the softening towards the 1/2 plateau, the constant velocity up to $H_{c7} = 116$ T, and the slope change around $H_{c8} = 127$ T. Supplementary Fig. 5(b) shows the magnetostriction measured for a single FBG setting, which survives even after the STC experiments. The slight difference might be due to the covering conditions with vacuum grease, which melts each time after the experiment. Again, the overall features are well reproduced; the rapid decrease at 80 T, the increase at $H_{c6} = 108$ T, the maximum at $H_{c7} = 116$ T, and the kink at $H_{c8} = 127$ T. The anomaly at $H_{c9} = 139$ T, which corresponds to the saturation of magnetization, is also reproduced for the ultrasound and magnetostriction measurements.

SUPPLEMENTARY NOTE 2: CRITICAL FIELDS

In the main text, we summarize the critical fields (H_{c6}, H_{c7}, H_{c8}, and H_{c9}) based on the experimental results in Fig. 2. In this section, we present the enlarged figure of these results

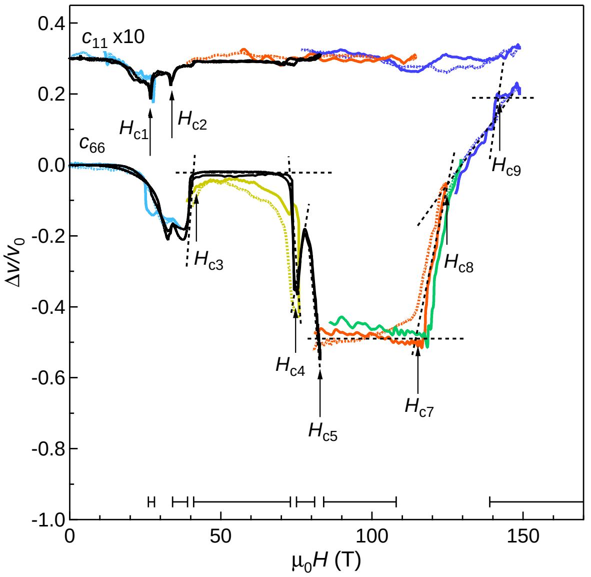

Supplementary Figure 6. Ultrasound results with the graphical definitions of the critical fields. Dashed lines are guide to the eye.

and explain the definition of the critical fields.

Supplementary Fig. 6 shows the relative changes of the sound velocity for the c_{11} and c_{66} mode, which are reproduced from Fig. 2(a) in the main text. The low-field phase boundaries (H_{c1}-H_{c5}) coincide with the earlier studies [5, 6]. H_{c7} is defined at the adrupt increase of the sound velocity of the c_{66} mode. The discontinuous change of the sound velocity and the hysteresis indicate that this transition is of the first order. H_{c8} is defined at the slope change (kink) of the sound velocity of the c_{66} mode. This phase boundary H_{c8} is indicated by the magnetostriction as well. H_{c9} is defined at the discontinuous increase of the sound velocity of the c_{66} mode. Above H_{c9}, the sound velocity change is saturated.

Supplementary Fig. 7 shows the magnetostriction data along the c axis, which are reproduced from Fig. 2(b) in the main text. The up- and down-sweep results are shifted for clarity and shown by the solid and dotted lines, respectively. We mainly use these results to support the phase boundaries determined from the ultrasound experiments. As explained in Supplementary Note 1, the experimental results obtained by the FBG are affected by the surrounding installation conditions and cannot be fully reproduced. In particular, the low-field phase boundaries (H_{c1}-H_{c5}) are challenging to define with the STC technique. In contrast, the reproducibility of H_{c6} and H_{c7} is quite good [see also Supplementary Fig. 5(b)]. At H_{c6}, the sample length discontinuously increases and shows a peak at H_{c7}. At H_{c8}, the

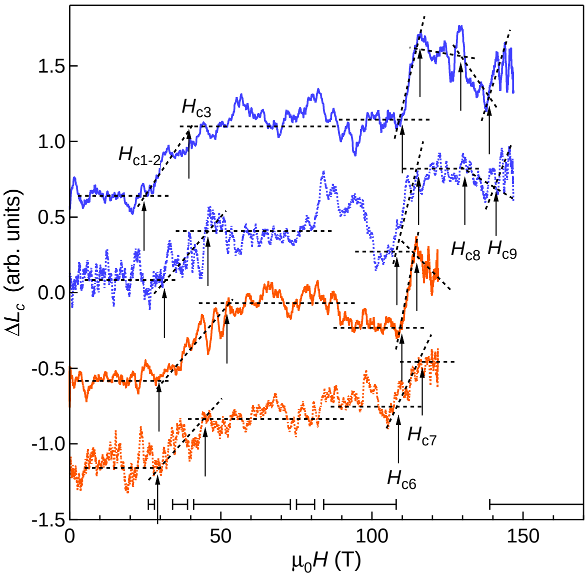

Supplementary Figure 7. Magnetostriction results with the graphical definitions of the critical fields. The up-sweep (solid lines) and down-sweep (dotted lines) results are separately shown. Dashed lines are guide to the eye.

length starts to decrease and take a minimum at H_{c9}. Although the anomalies at H_{c8} and H_{c9} are relatively weak, they are reproduced for the up and down sweeps.

These phase boundaries are systematically determined from the experimental observations (ultrasound, magnetostriction, magnetization). The critical fields summarized in Table I in the main text are estimated from each techniques independently. The error ranges are estimated by considering the ambiguity of the anomaly, reproducibility of the critical field, and typical error of the magnetic field value.

SUPPLEMENTARY NOTE 3: EXTENT OF THE PERIOD-10 SUPERSOLID PHASE

The extent of the period-10 SSP in the phase diagram (Fig. 3) in the main text is extremely narrow and (coincidentally) terminates around the experimentally relevant value of $J'/J = 0.63$. In Supplementary Fig. 8, we present additional results for lower values of J'/J, which show that the extent of the period-10 SSP phase increases with decreasing J'/J.

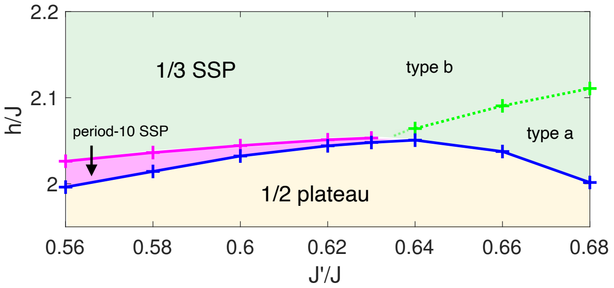

Supplementary Figure 8. iPEPS phase diagram around the period-10 SSP including lower values of J'/J down to 0.56 (with $D = 8$ and cluster optimization). The extent of the period-10 SSP gets considerably larger at lower values of J'/J.

SUPPLEMENTARY NOTE 3: MAGNETIC CONTRIBUTIONS TO THE c_{66} MODE

In this section, we discuss the relation between the first and the second derivatives of the magnetic energy with respect to the displacement ε associated with the mode probed by c_{66}. Without the magnetic contribution, and up to a constant, the structural energy is minimal for $\varepsilon = 0$, and its expansion in ε reads:

$$E_{struc} = b\varepsilon^2 + c\varepsilon^3$$

where b describes the harmonic potential and c the cubic anharmonic contribution.

As shown by iPEPS calculations, the magnetic contribution to the energy has both linear and quadratic contributions:

$$E_{mag} = a\varepsilon + b'\varepsilon^2$$

with $a = dE_{mag}/d\varepsilon$ and $b' = (1/2)d^2E_{mag}/d\varepsilon^2$.

Because of the linear term of E_{mag}, the minimum of the total energy $E_{tot} = E_{struc} + E_{mag}$ occurs at a non-zero value ε_0 which is solution of the equation

$$\frac{dE_{tot}}{d\varepsilon} = a + 2(b + b')\,\varepsilon + 3\,c\,\varepsilon^2 = 0.$$

If ε_0 is very small, which will be the case if $a, c \ll b$, then it is given by

$$\varepsilon_0 \simeq -\frac{a}{2(b + b')}.$$

Now let us expand the total energy around the new minimum by setting $\varepsilon = \varepsilon_0 + \delta$. Up to the second order in δ, we get:

$$\begin{aligned} E_{tot}(\delta) &= a\varepsilon_0 + (b+b')\varepsilon_0^2 + c\varepsilon_0^3 \\ &\quad + \left(a + 2(b+b')\varepsilon_0 + 3c\varepsilon_0^2\right)\delta \\ &\quad + \left(b + b' + 3c\varepsilon_0\right)\delta^2. \end{aligned}$$

The coefficient of the linear term vanishes because of the condition satisfied by ε_0, and, in the limit of small ε_0, the coefficient of the quadratic term is given by

$$b + b' - \frac{3c}{2(b+b')}a.$$

As anticipated, it contains a contribution proportional to b', hence to $d^2E_{mag}/d\varepsilon^2$, and a contribution proportional to a, hence to $dE_{mag}/d\varepsilon$. The contribution of the first derivative comes from the anharmonicity - its coefficient is proportional to c.

---

[1] T. Nomura, A. Hauspurg, D. I. Gorbunov, A. Miyata, E. Schulze, S. A. Zvyagin, V. Tsurkan, Y. H. Matsuda, Y. Kohama, and S. Zherlitsyn, Ultrasound measurement technique for the single-turn-coil magnets, Rev. Sci. Instrum. **92**, 063902 (2021).

[2] A. Ikeda, T. Nomura, Y. H. Matsuda, S. Tani, Y. Kobayashi, H. Watanabe, and K. Sato, High-speed 100 MHz strain monitor using fiber bragg grating and optical filter for magnetostriction measurements under ultrahigh magnetic fields, Rev. Sci. Instrum. **88**, 083906 (2017).

[3] A. Ikeda, Y. H. Matsuda, and K. Sato, Two spin-state crystallizations in $LaCoO_3$, Phys. Rev. Lett. **125**, 177202 (2020).

[4] R. Schönemann, G. Rodriguez, D. Rickel, F. Balakirev, R. D. McDonald, J. A. Evans, B. Maiorov, C. Paillard, L. Bellaiche, A. V. Stier, M. B. Salamon, K. Gofryk, and M. Jaime, Magnetoelastic standing waves induced in UO_2 by microsecond magnetic field pulses, Proc. Natl. Acad. Sci. **118**, e2110555118 (2021).

[5] Y. H. Matsuda, N. Abe, S. Takeyama, H. Kageyama, P. Corboz, A. Honecker, S. R. Manmana, G. R. Foltin, K. P. Schmidt, and F. Mila, Magnetization of $SrCu_2(BO_3)_2$ in ultrahigh magnetic fields up to 118 T, Phys. Rev. Lett. **111**, 137204 (2013).

[6] M. Jaime, R. Daou, S. A. Crooker, F. Weickert, A. Uchida, A. E. Feiguin, C. D. Batista,

H. A. Dabkowska, and B. D. Gaulin, Magnetostriction and magnetic texture to 100.75 tesla in frustrated $SrCu_2(BO_3)_2$, Proc. Natl. Acad. Sci. **109**, 12404 (2012).
